# Protective activity ethanol extract of the fruits of *Illicium verum* against atherogenesis in apolipoprotein E knockout mice

**DOI:** 10.1186/s12906-015-0750-0

**Published:** 2015-07-15

**Authors:** Sun Haeng Park, Yoon-Young Sung, Kyoung Jin Nho, Ho Kyoung Kim

**Affiliations:** Mibyeong Research Center, Korea Institute of Oriental Medicine (KIOM), 1672 Yuseong-daero, Yuseong-gu, Daejeon, 305-811 South Korea

**Keywords:** Atherosclerosis, *ApoE*-knockout mice, Inflammation, Hypercholesterolemia, *Illicium verum*

## Abstract

**Background:**

*Illicium verum* Hook. fil. Illiciaceae (*Illicium v*.) has been traditionally used in herbal medicine for treating many inflammatory diseases, including skin inflammation and rheumatism. We investigated its use as a preventive agent against inflammatory and vascular diseases in a murine model of atherosclerosis using apolipoprotein E-knockout (*ApoE*^−/−^) mice fed on a high-fat diet (HFD).

**Methods:**

We investigated the effect of *Illicium v*. on cytotoxicity, NF-κB activity, and adhesion molecule expression in TNF-α – stimulated HASMCs (Human Aortic smooth muscle cells). *ApoE*^−/−^mice, fed a HFD and treated daily for 12 weeks by oral administration of either *Illicium v*. (100 or 200 mg/kg) or atorvastatin (10 mg/kg), were evaluated for atherosclerotic lesions and inflammatory responses by performing Oil red O and iNOS staining, respectively. Expression of inflammatory cytokines (i.e., NF-κB, TNF-α, IL-1β, COX, IκB-α, Iκκ-α/β) and adhesion molecules in the aorta were measured by western blot analysis.

**Results:**

In TNF-α-stimulated HASMCs, *Illicium v*. treatment decreased NF-κB transcriptional activity, and NF-κB protein levels were reduced in a dose-dependent manner over a range of 10–100 μg/mL *Illicium v*. Also, *Illicium v*. attenuated the expression of adhesion molecules that are responsible for inflammation in these cells. In animal experiments, treatment with *Illicium v*. or atorvastatin counteracted the characteristic changes in body weight, blood pressure, and lipid levels seen in HFD-fed *ApoE*^−/−^ mice. In addition, *Illicium v*. treatment reduced aortic atherosclerotic plaque lesions and the immunoreactivity of iNOS activation. The aortic expression of inflammatory adhesion molecules and cytokines (TNF-α, IL-1β, NF-κB, COX, IκB-α, Iκκ-α/β), which is characteristic of HFD-fed *ApoE*^−/−^ mice, was attenuated by 12-week treatment with daily oral administration of *Illicium v*. or atorvastatin, and the most potent effect was seen with the herbal tincture.

**Conclusions:**

The beneficial effects of *Illicium v*. are consistent with a significant decrease in the iNOS-mediated inflammatory response, resulting in reduction of inflammation-associated gene expression. Treatment with *Illicium v*. may be the basis of a novel therapeutic strategy for hyperlipidemia-atherosclerosis.

## Background

Hyperlipidemia plays an important role in the pathogenesis of atherosclerosis in prehypertension [13]. Many studies have demonstrated that systemic inflammation and the immune system play a central role in atherogenesis. Strong dependence of the atherosclerotic process on both a state of continuous low-grade inflammation and presence of lipid abnormalities gave impetus in the field to examine the association between hyperlipidemia and inflammatory status [26]. Recent studies in hyperlipidemic mice have demonstrated that anti-inflammatory compounds prevent development of atherosclerosis without altering blood lipid profiles [2, 12, 22]. Furthermore, anti-inflammatory drugs there are other agents that can reduce the occurrence of atherosclerosis without altering blood lipid profiles in this murine model, indicating that anti-inflammatory compounds may be used as therapeutic drugs for the treatment of hyperlipidemia-atherosclerosis [16].

Monocytes and macrophages infiltrate areas of atherosclerotic lesions and increase expression of adhesion molecules and inflammation-related genes, such as ICAM, VCAM, NF-κB, and iNOS, that are important for development and progression of atherosclerosis [3, 12]. In particular, large amount of locally released NO has been linked to generation of harmful oxidative products, such as peroxynitrite, that have been implicated in iNOS-mediated development of atherosclerosis [31]. The hypothesis that iNOS plays a causative role in the progression of atherosclerosis is supported by the observation that atherosclerotic lesions are diminished in *iNOS*/*ApoE* double-knockout mice relative to *ApoE*^−/−^ mice [15]. Furthermore, iNOS expression is activated by NF-κB, which is responsive to inflammation and oxidative stress [23]. Therefore, understanding mechanisms of iNOS regulation may provide new targets for the treatment of hyperlipidemia-atherosclerosis.

*Illicium verum* Hook. fil. Illiciaceae (*Illicium v*.), is widely used for culinary and medicinal purposes, and it is taken as a natural remedy by many patients. The fruit of the plant has been used in traditional medicine for treatment of stomach aches, vomiting, rheumatic pain, insomnia, and skin inflammation [11, 18, 27, 30], and it has been reported to have beneficial anti-inflammatory effects. These effects have been shown with ethanolic *Illicium v*, extracts to be mediated by suppressing the expression of TNF-α- and IFN-γ-stimulated chemokine and cytokine expression *in vitro* and *in vivo* [20, 27, 28]. *Illicium v*. contains ten main chemical entities (i.e., linoleic acid, palmitic acid, *trans*-anethole, anisaldehyde, estragole, limonens, (*Z*)-anethole, pinene, β-phellandraene, and α-terpineol) that have various anti-inflammatory pharmacological effects [7, 14, 25, 28].

Given the known inflammatory component in atherosclerosis, it is perhaps surprising that *Illicium v*. has not yet been tested as a potential treatment for atherosclerosis or hyperlipidemia in an animal model. It is reasonable to hypothesize that *Illicium v*. might be efficacious because of its demonstrated anti-inflammatory action in other settings. We hypothesized that *Illicium v*. would be active as an anti-inflammatory agent in *ApoE*^−/−^ mice and would inhibit atherosclerosis in these animals when they are fed a high-fat diet (HFD), which is an accepted mechanism-based animal model for the disease. A number of parameters were investigated *in vivo*, such as body weight, blood lipid levels, and blood pressure, during HFD-induced atherosclerosis to evaluate activity of *Illicium v*. Effects of the extract on expression of atherosclerosis-related adhesion molecules and anti-inflammatory signaling in human smooth muscle cells (HASMCs), as well as in *ApoE*^−/−^ mouse aortas, were assessed.

## Methods

### Cell culture

Cryopreserved HASMC were purchased from ScienCell Research Laboratory (San Diego, CA, USA). Cells were cultured as monolayers in smooth muscle cell medium (ScienCell) containing essential and nonessential amino acids, vitamins, organic and inorganic compounds, hormones, growth factors, trace minerals, and 2 % (*v*/*v*) fetal bovine serum at 37 °C in a humidified 5 % CO_2_ atmosphere. Cells from passages 2 to 6 were used in this study.

### Experimental animals and diet

Male *ApoE*^−/−^ mice in a C57BL/6 N background (6 weeks of age) were from the Jackson Laboratory (Bar Harbor, ME, USA). They were housed under diurnal lighting conditions and allowed food and tap water ad libitum. All experimental protocols involving use of animals were conducted in accordance with the National Institutes of Health guidelines and approved by the Committee on Animal Care of KIOM.

The mice fed on a High Fat diet (45 % of total calories from fat; 0.15 % cholesterol; Research Diet, New Brunswick, NJ, USA) were divided into five groups: control (chow diet, *n* = 8), HFD (High Fat Diet, *n* = 8), *Illicium v*. 100 mg/kg treatment (HFD with *Illicium v*. 100 mg/kg, *n* = 8), *Illicium v. 2*00 mg/kg (HFD with *Illicium v*. 200 mg/kg, *n* = 8), and atorvastatin 10 mg/kg (HFD with atorvastatin 10 mg/kg, *n* = 8). Daily treatments were given orally over a 12-week period, during which experimental animals were fed a HFD.

### Preparation of *Illicium verum* extract

*Illicium v.* material was purchased from Omniherb Co. (Yeongcheon, South Korea) and was authenticated based on its microscopic and macroscopic characteristics by the Classification and Identification Committee of the KIOM. A voucher specimen has been deposited at the herbarium of the Basic Herbal Medicine Research Group at KIOM. Dried fruit (300 g) was extracted twice with 70 % (*v*/*v*) ethanol (with a 2-h reflux). The extract was concentrated under reduced pressure at 40 °C with a rotary evaporator. The decoction was filtered, lyophilized, and stored at 4 °C until use (Sung et al. [27]). The yield of the dried extract from the starting crude materials was approximately 15.73 % (*w/w*). The lyophilized powder was dissolved in 0.05–0.1 % dimethyl sulfoxide and then filtered through a 0.22 μm syringe filter to create a stock solution.

### Cell viability

A standard assay based on the tetrazole (3-(4,5-dimethylthiazol-2-yl)-2,5-diphenyltetrazolium bromide (MTT) was used to assess cell viability. Briefly, HASMCs were seeded in 96-well microtiter plates at a density of 1 × 10^5^ cells/well. They were treated with various concentrations of *Illicium v.* (10, 50, or 100 μg/mL) for 24 h. Subsequently, 100 μL of 5 mg MTT/mL in PBS was added to each well, and the plates were incubated at 37 °C for 4 h before adding 200 μL DMSO to dissolve formazan crystals. Absorbance was measured at 540 nm by spectrophotometry.

### *NF-*κ*B* activity assay

Activity of NF-κB was measured by luciferase reporter assays in HASMC. Cells in 12-well plates were cotransfected with a firefly luciferase gene tagged with renilla luciferase and pGL4.32-NF-κB, using the FuGENE HD reagent (Invitrogen; Carlsbad, CA, USA). Medium was replaced with fresh medium after 6 h. At 24 h post transfection, cells were stimulated with TNF-α (10 ng/mL, R&D Systems Inc.; St. Louis, Mo, USA) in the presence of 10, 50, or 100 μg *Illicium v.*./mL. Luciferase activity was assayed 24 h later using a dual-luciferase reporter assay system (Promega; Madison, Wl, USA).

### Measurement of serum markers

Blood was collected from the aorta under light anesthesia and stored on ice for 30 min before centrifugation at 13,000 rpm at 4 °C for 10 min, and the serum was separated and kept at −80 °C until it was thawed for the assay. Serum levels of total cholesterol, high-density lipoprotein (HDL), low-density lipoprotein (LDL), triglycerides, glucose, alanine aminotransferase (ALT), aspartate aminotransferase (AST), and creatinine were measured with an automatic analyzer.

### Blood pressure measurement

Blood pressure was monitored using a noninvasive tail-cuff CODA^TM^ system (Kent Scientific; Torrington, CT, USA) as previously described [17].

### Histopathology

Mice were deeply anesthetized with sodium thiopental and subsequently the tissue was removed and then washed with cold PBS and followed fixation with 4 % (*w/v*) paraformaldehyde. The aorta of each mouse was then removed and further fixed in the same solution for 24 h at 4 °C. Fixed aortas were frozen and stored at −80 °C. Sections of 10-μm thickness were sliced from frozen tissue and then immunostained with antibodies against iNOS(Thermo Scientific; Rockford, IL USA). After additional incubation with secondary antibody, sections were reacted with 3-amino-9-ethylcarbazole chromogen (Vector Laboratories; Burlingame, CA, USA). Reactions with 3,3′ diaminobenzidine substrate (Vector Laboratories) were performed for color development, and the stained sections were then analyzed by light microscopy.

### Western blot analysis

Proteins were isolated from aortic tissue according to standard techniques [4], separated by 10 % sodium dodecyl sulfate-polyacrylamide gel electrophoresis and then transferred onto nitrocellulose membranes (Amersham Biosciences; Piscataway, NJ, USA). Blots were probed with primary antibodies directed against VCAM-1 or NF-κB (Santa Cruz Biotechnology; San Cruz, CA, USA), or against ICAM-1, E-selectin, TNF-α, IL-1β, COX, IκB-α, or Iκκ-α/β, iNOS (Cell Signaling Technology; Beverly, MA, USA). This was followed by incubation with secondary antibody conjugated to horseradish peroxidase (Cell Signaling). Bound secondary antibody was detected by chemiluminescence generated by peroxidase reaction, measured with an ImageQuant LAS 4000 apparatus (GE Healthcare Life Sciences; Buckinghamshire, UK). Membranes were reprobed with an anti-β-actin antibody (Sigma-Aldrich Chemical Co.; St. Louis, MO, USA) as internal sample control.

### Data analysis

The data are expressed as mean values ± SEM. Statistical comparisons were performed using analysis of variance for repeated measures, followed by Sigmastat statistical program Version 11.2 (Systat Software, San Jose, CA, USA). Data were analyzed statistically using two-way ANOVA via Tukey’s post hoc comparison when comparing more than two groups. A value of *P* <0.05 was considered to be statistically significant.

## Results

### *Illicium v.* effects on TNF-α-stimulated HASMCs

**Fig. 1:**
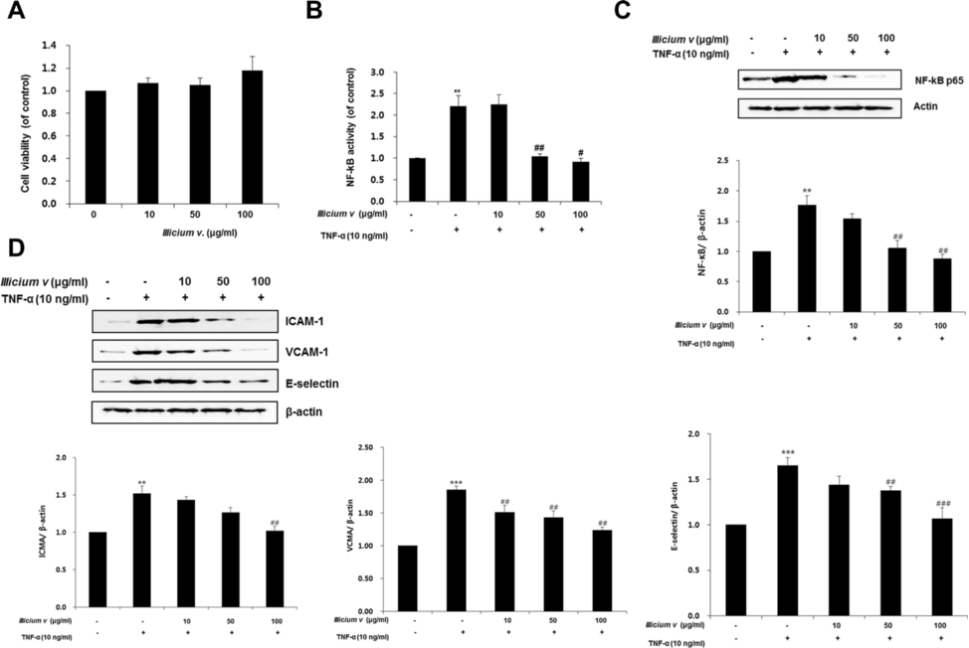
*Illicium v.* inhibits inflammatory gene expression in TNF-α-stimulated human aortic smooth muscle cells. **a** Human aortic smooth muscle cells (HASMC) were treated with or without *Illicium v.* (10–100 μg/mL) for 24 h. After incubation, cell viability was determined using the MTT assay. **b** HASMCs were transiently transfected with pGL4.32-NF-κB, a renilla luciferase control reporter vector, and then treated with or without *Illicium* v. (10–100 μg/mL), followed by incubation with 10 ng/mL TNF-α for 24 h. NF-κB transcription activity was determined using a luciferase reporter assay. **c** Protein samples from HASMCs, treated with or without *Illicium v.* (10–100 μg/mL) and 10 ng/mL TNF-α for 24 h, examined by western blot analysis for NF-κB protein expression. **d** Protein expression of adhesion molecules (ICAM, VCAM, and E-selectin). All data represent mean values ± SEM. **P < 0.01, ***P < 0.001 versus control; ^#^ *P* < 0.05, ^##^ *P* < 0.01, ^###^ *P* < 0.001 versus HFD

Cytotoxic effect of *Illicium v.* was assessed at different concentrations (0, 10, 50, or 100 μg/mL) by MTT assay in HASMCs. At concentrations in this range, *Illicium v.* did not cause any apparent cytotoxicity (Fig. 1a). We evaluated the ability of *Illicium v.* to decrease pro-inflammatory NF-κB activity in TNF-α-stimulated HASMCs. The TNF-α-stimulated NF-κB transcription activity (22.03 ± 0.25 vs. control group) was markedly reversed by *Illicium v.* in a dose-dependent manner (Fig. 1b). The activity was decreased by 50 and 100 μg/mL *Illicium v.* decreased to 10.39 ± 0.06 and 9.17 ± 0.07, respectively (*P* < 0.01, *P* < 0.05 vs. TNF-α-stimulated group). Furthermore, NF-κB protein level was decreased by *Illicium v.* in TNF-α-stimulated HASMCs (Fig. 1c). Western blot analysis revealed that adhesion molecule (ICAM, VCAM, and E-selectin) expression was induced in the TNF-α-stimulated group. *Illicium v.* significantly antagonized TNF-α-induced ICAM, VCAM, and E-selectin expression (Fig. 1d). Transcriptional activity of many of these inflammatory cytokines and adhesion molecules is controlled by NF-κB. These results show that *Illicium v.* suppresses production of inflammatory mediators *in vitro.*

### Body weight and blood pressure in treated and untreated HFD-fed ApoE^−/−^ mice

**Fig. 2:**
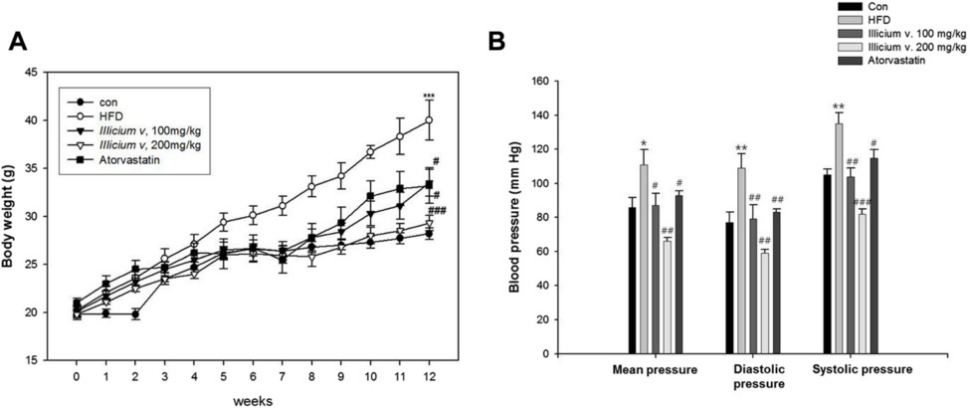
*Illicium v.* results in improved body weight and blood pressure in high-fat diet-fed *ApoE* ^−/−^ mice. **a** Body weight ratio was measured during the 0–12 week feeding regimen. **b** Blood pressure (diastolic and systolic) ratio was measured at the end of the 12-week feeding regimen. All data represent mean values ± SEM. *P < 0.05, **P < 0.01, ***P < 0.001 versus control; ^#^ *P* < 0.05, ^##^ *P* < 0.01, ^###^ *P* < 0.001 versus HFD

Body weights of *ApoE*^*−/−*^ mice fed on a HFD for 12 week were higher than those in normal diet-fed mice (control) (28.2 ± 0.60 vs. 40.0 ± 2.10 g, *P* < 0.001). However, the *Illicium v.* 100 and 200 mg/kg group mean body weights were significantly lower than those of mice in the HFD group (33.5 ± 1.40 and 29.3 ± 0.78 g vs. HFD group, *P* < 0.01 and *P* < 0.001, respectively). This was similar to the effect of atorvastatin (33.2 ± 1.85 vs. HFD group, *P* < 0.05; see Table 1 and Fig. 2a). Next, we confirmed that *Illicium v.* reduced blood pressure. During the course of the experiment, blood pressure in the HFD group was significantly higher than that in the control group (86 ± 5.59 vs. 111 ± 8.80 mmHg, *P* < 0.05). In contrast, the blood pressure was significantly lower in the groups treated with either *Illicium v.* at 100 mg/kg (87 ± 7.13 mmHg, *P* < 0.05) or at 200 mg/kg (66 ± 2.16 mmHg, *P* < 0.01) or in the atorvastatin group (93 ± 2.66 mmHg, *P* < 0.05) than HFD group. Moreover, diastolic and systolic blood pressure levels were significantly reduced in the *Illicium v.* and atorvastatin treatment groups (Table 1 and Fig. 2b).

**Table 1:** Physiological parameters

		Blood pressure (mm Hg)		
	Body weight (g)	Mean pressure	Diastolic	Systolic
Con	28.2 ± 0.60	86 ± 5.559	77 ± 6.19	105 ± 3.51
HFD	40.0 ± 2.10***	111 ± 8.80*	109 ± 8.41**	135 ± 6.52**
*Illicium v.,* 100 mg/kg	33.5 ± 1.40^#^	87 ± 7.13^#^	79 ± 8.42^##^	104 ± 4.91^##^
*Illicium v.,* 200 mg/kg	29.3 ± 0.78^###^	66 ± 2.16^##^	59 ± 2.04^##^	82 ± 2.83^###^
Atorvastatin	33.2 ± 1.85^#^	93 ± 2.66^#^	83 ± 1.94^##^	115 ± 4.84^#^

Abbreviations: *Con*, control; *HFD*, high-fat diet

All data represent mean values ± SEM. (*n* = 8)

^*^ *P* < 0.05, ^**^ *P* < 0.01 and ^***^ *P* < 0.001, versus chow diet fed *ApoE* ^*−/−*^ mice (control)

^#^ *P* < 0.05, ^##^ *P* < 0.01 and ^###^ *P* < 0.001, versus HFD-fed *ApoE* ^*−/−*^ mice (HFD)

### Blood lipid levels

*Illicium v.-* or atorvastatin-treated HFD-fed *ApoE*^*−/−*^ mice showed significantly decreased total and LDL cholesterol levels (*P* < 0.05 and *P* < 0.01 vs. HFD group; Table 2). Moreover, glucose levels were decreased in *Illicium v.* 50 mg/kg and 100 mg/kg groups (245.67 ± 14.54 and 215.33 ± 10.23 vs. HFD group, *P* < 0.01 and *P* < 0.001, respectively). Potential toxicity did not differ among mice treated with *Illicium v.* or atorvastatin, at inhibitory concentrations of both agents.

**Table 2:** Lipid parameters

			*Illicium verum*		
	Con	HFD	100 mg/kg	200 mg/kg	Atorvastatin
T-chole (mg/dL)	502.40 ± 22.79	1112.00 ± 120.77***	635.50 ± 106.12^#^	608.00 ± 49.50^##^	655.25 ± 60.80^#^
LDL (mg/dL)	79.60 ± 7.86	244.00 ± 48.45***	134.17 ± 22.74^#^	108.33 ± 15.19^##^	98.75 ± 17.05^#^
HDL (mg/dL)	16.80 ± 2.04	9.83 ± 1.30**	8.50 ± 1.46	11.44 ± 1.71	12.25 ± 2.88
TG (mg/dL)	41.00 ± 6.16	34.60 ± 3.29	33.80 ± 4.77	38.71 ± 6.35	34.50 ± 7.37
Glucose (mg/dL)	262.80 ± 9.82	418.67 ± 26.47**	245.67 ± 14.54^##^	215.33 ± 10.23^###^	371.25 ± 40.82
AST (U/L)	110.20 ± 14.88	175.17 ± 28.16	196.50 ± 49.89	162.67 ± 31.01	123.50 ± 19.75
ALT (U/L)	43.40 ± 2.70	100.67 ± 27.88	60.33 ± 8.06	46.78 ± 2.43^#^	49.25 ± 4.50
Creatinine (mg/dL)	0.17 ± 0.01	0.18 ± 0.01	0.19 ± 0.02	0.13 ± 0.02	0.23 ± 0.02

Abbreviations: *Con*, control; *HFD*, high-fat diet; *T-chole*, total cholesterol; *LDL*, low density lipoprotein; *HDL*, high density lipoprotein; *TG*, triglyceride; *AST*, aspartate aminotransferase; *ALT*, alanine aminotransferase

All data represent mean values ± SEM. (*n* = 8)

^**^ *P* < 0.01, ^***^ *P* < 0.001, versus chow diet fed *ApoE* ^*−/−*^ mice (control)

^#^ *P* < 0.05, ^##^ *P* < 0.01, and ^###^ *P* < 0.001, versus HFD-fed *ApoE* ^*−/−*^ mice (HFD)

### *Illicium v.* attenuates formation of atherosclerotic plaque lesions

**Fig. 3:**
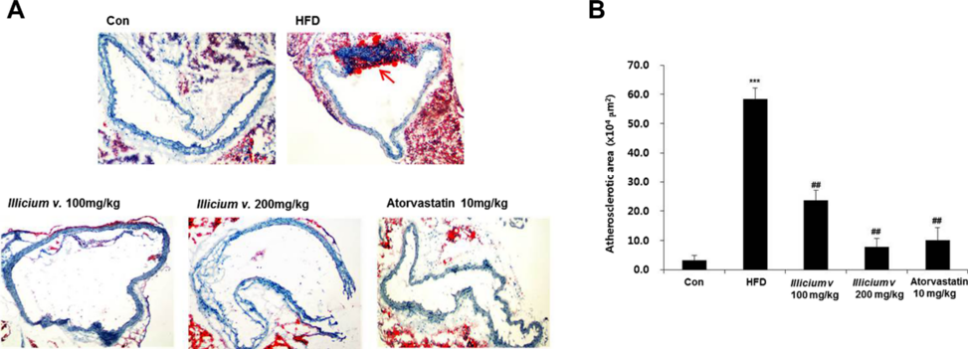
*Illicium v.* reduces atherosclerotic lesions in high-fat diet-fed *ApoE* ^−/−^ mice. The *ApoE* ^−/−^ mice were fed high-fat diet (HFD) with or without *Illicium v.* (100 or 200 mg/kg) for 12 week. **a** Photographs of Oil red O staining in the aorta, showing atherosclerotic lesions from each experimental group. **b** Quantification of Oil red O-stained atherosclerotic lesion areas from each group. All data represent mean values ± SEM. ***P < 0.001 versus control; ^##^ *P* < 0.01 versus HFD

We investigated whether *Illicium v.* exerts an anti-atherosclerotic effect in HFD-fed *ApoE*^*−/−*^ mice by quantifying atherosclerotic plaque lesional areas. Consecutive cross sections of aortas stained with Oil red O revealed induction of plaque formation. The mice that were fed a HFD for 12 weeks exhibited significantly increased aortic atherogenic plaque areas as compared to mice fed control diet (3.20 ± 1.69 vs. 58.36 ± 3.92 × 10^4^ μm^2^). This increase was significantly antagonized by dose-dependent supplementation with *Illicium v.* (23.69 ± 3.32 and 7.87 ± 2.89 × 10^4^ μm^2^) or atorvastatin (10.0 ± 4.41 × 10^4^ μm^2^, *P* < 0.01 vs. HFD group) (Fig. 3a and b).

### *Illicium v.* attenuates iNOS expression in the aorta

**Fig. 4:**
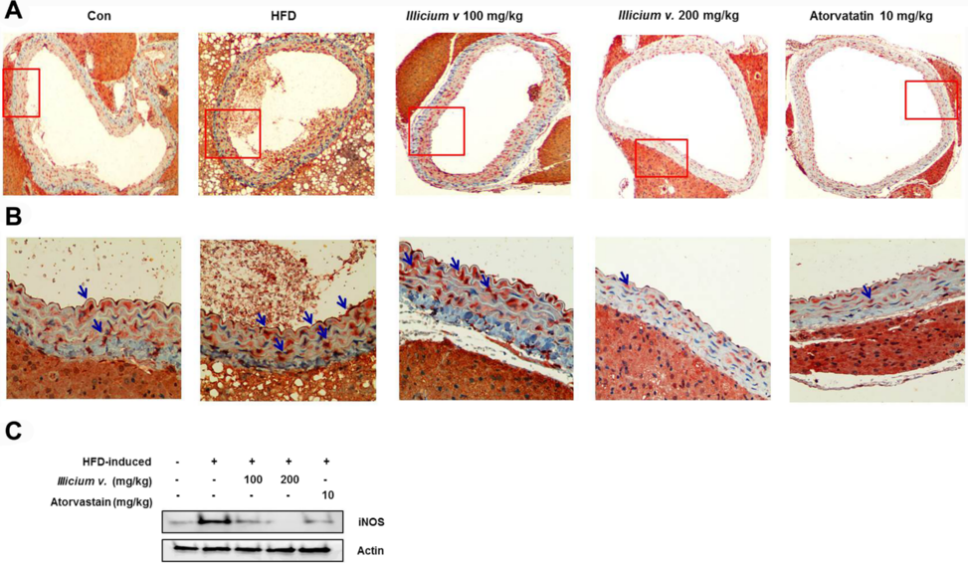
Representative histological cross section of the aorta in high-fat diet-fed *ApoE* ^−/−^ mice. The *ApoE* ^−/−^ mice were fed high-fat diet (HFD) with or without *Illicium v.* (100 or 200 mg/kg) for 12 week. **a** and **b** Aorta tissue samples were excised, fixed in 4 % formaldehyde, embedded in paraffin and then sectioned. Immunohistochemistry of sections stained for iNOS (A, magnification × 200; B, ×400). **c** Protein levels of iNOS were determined by western blot analysis

To explore action mechanisms of *Illicium v.* effects in hyperlipidemia with atherosclerosis, we studied aortic iNOS expression levels in the mouse model. Many iNOS cells were observed in the HFD-fed *ApoE*^*−/−*^ mice. *Illicium v.* 200 mg/kg treatment resulted in complete reduction of iNOS activation in HFD-fed *ApoE*^*−/−*^ mice (Fig. 4a and b). Furthermore, iNOS expression was decreased by 200 mg/kg Illicium v. in protein of aortic tissue (Fig. 4c). These data suggest an iNOS-dependent mechanism.

### *Illicium v.* inhibits production of inflammatory mediators in the aorta

**Fig. 5:**
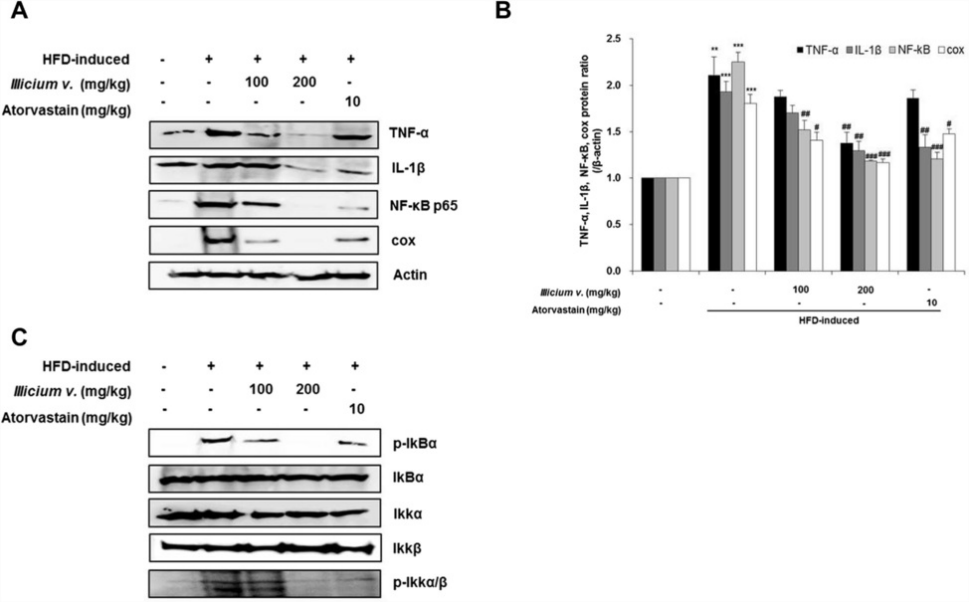
*Illicium v.* decreases expression levels of NF-κB and other inflammatory genes in aortas from high-fat diet-fed *ApoE* ^−/−^ mice. **a** Expression levels of TNF-α, IL-1β, NF-κB, and COX were determined by western blot analysis. **b** Relative abundance of protein was calculated by ratio for expressed TNF-α, IL-1β, NF-κB, and COX. **c** Total protein expression and phosphorylation of IκB-α and Iκκ-α/β were detected by western blot analysis. All data represent mean values ± SEM. ***P < 0.001 versus control; ^#^ *P* < 0.05, ^##^ *P* < 0.01, ^###^ *P* < 0.001 versus HFD

Previously published studies on sepsis show that high iNOS expression is associated with inflammatory mediators, such as TNF-α, IL-1β, NF-κB, and COX. These mediators also play an important role in the development and progression of atherosclerosis. We examined the effects of *Illicium v.* on expression of these proteins in aortic tissues. Levels of secreted TNF-α, IL-1β, NF-κB, and COX proteins were significantly increased in aortic tissues from HFD-fed *ApoE*^*−/−*^ mice, and these increases were antagonized by supplementation with *Illicium v.* or atorvastatin (*P* < 0.05, <0.01, and <0.001 vs. HFD group) (Fig. 5a and b). In addition, IκB-α/Iκκ-α/β expression was also increased in aortic tissue from HFD-fed *ApoE*^*−/−*^ mice, and these increases were antagonized by *Illicium v.* or atorvastatin (Fig. 5c). These results suggest that *Illicium v.* regulates inflammatory gene expression via mediation of reduced iNOS activation.

### *Illicium v.* inhibits expression levels of adhesion molecules in the aorta

**Fig. 6:**
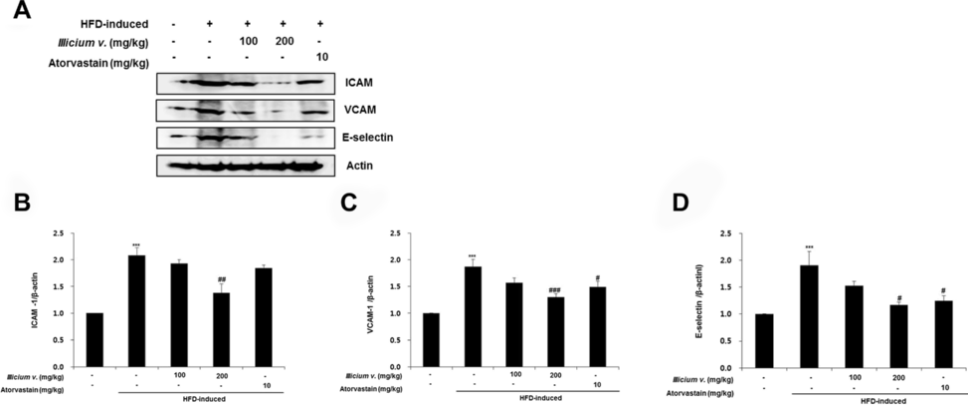
*Illicium v.* suppresses adhesion molecule expression in aortas from high-fat diet-fed *ApoE* ^−/−^ mice. **a** Protein levels of ICAM-1, VCAM-1, and E-selectin were determined by western blot analysis. **b** Relative abundance of protein was calculated by ratio for ICAM-1, VCAM-1, and E-selectin. All data represent mean values ± SEM. ***P < 0.001 versus control; ^#^ *P* < 0.05, ^##^ *P* < 0.01, ^###^ *P* < 0.001 versus HFD

To determine involvement of adhesion molecule expression, including ICAM-1, VCAM-1, and E-selectin, in the anti-inflammatory activities of *Illicium v.*, protein levels were examined in western blots (Fig. 6a and b). Levels of ICAM-1, VCAM-1, and E-selectin were higher in aortas of untreated HFD-fed *ApoE*^*−/−*^ mice than normal controls. Treatment with *Illicium v.* or atorvastatin resulted predominantly in a dose-dependent downregulation of ICAM-1, VCAM-1, and E-selectin expression levels in aortas of HFD-fed *ApoE*^*−/−*^ mice. These results indicate that *Illicium v.* efficiently lowers adhesion molecule protein levels in hyperlipidemia-atherosclerosis.

## Discussion

We have defined mechanisms involved in the beneficial effects of *Illicium v.* on atherosclerosis plaque lesions by investigating effects of the agent on anti-inflammatory responses in HASMCs *in vitro*, and in aortas of HFD-fed *ApoE*^*−/−*^ mice *in vivo*. We report that *Illicium v.* decreases characteristic changes in body weight, blood pressure, and lipid levels in HFD-fed *ApoE−/−* mice. In addition, *Illicium v.* strongly attenuates atherosclerotic plaque lesions in the aortas of those mice, and it decreases immunoreactivity of iNOS. Furthermore, significantly lower levels of TNF-α, IL-1β, NF-κB, COX, IκB-α, Iκκ-α/β and adhesion molecules were seen with *Illicium v.* treatment. The results clearly demonstrate beneficial effects of *Illicium v.* on pathogenic atherosclerotic plaques and subsequent vascular impairment by vascular inflammation.

Pathogenesis of hyperlipidemia-atherosclerosis involves a chronic inflammatory state characterized by lipid deposition, accumulation of macrophages, and vascular smooth muscle cell proliferation in vascular walls [24, 29]. These are all well-known events in the development of the disease. Additionally, anti-inflammatory compounds have been found to effectively inhibit atherosclerotic plaque formation in a hyperlipidemic animal model [22]. Therefore, an iNOS imbalance is regarded as an important cause for the development of inflammatory-related diseases. Abnormal activation of iNOS and enhanced levels of inflammatory proteins, such as NF-κB, TNF-α, and certain adhesion molecules, are observed in the coronary plaques of patients [8].

Overexpression of iNOS has a causal effect on the development of atherosclerosis. This has been previously established by showing that atherosclerotic lesions are attenuated when iNOS is knocked out in *ApoE*^*−/−*^ mice, creating the double knock-out strain. Elucidation of factors that modulate aggregation of atherosclerotic plaques and adhesion molecule expression is crucial for intervention in hyperlipidemia-atherosclerosis [1, 9]. Several lines of evidence suggest that iNOS effects in vascular inflammation play a major role in hyperlipidemia pathogenesis, and they also influence various pathogenic processes implicated in hyperlipidemia [6]. Previous studies in hyperlipidemic murine models have demonstrated that lack of iNOS leads to a dramatic reduction in the size of atherosclerotic lesions. Furthermore, iNOS regulates expression of inflammatory cytokines, such as IL-1β, Iκκ-α/β, and TNF-α, in the aorta [10, 12]. Therefore, modulating the function or levels of proteins involved with iNOS activity in the aorta will likely affect vascular inflammation and, ultimately, pathogenic processes in hyperlipidemia. In our study with HFD-fed *ApoE*^*−/−*^ mice, *Illicium v.* was found to dramatically reduce the levels of inflammatory cytokines and adhesion molecules, subsequently improving atherosclerotic lesions. Moreover, *Illicium v.* reduced NF-κB expression. Therefore, the mechanism(s) of *Illicium v.* may reveal potential new targets for hyperlipidemia-atherosclerosis therapy, through its inhibitory activities against atherosclerotic lesion growth/aggregation and/or inflammatory responses. Moreover, *Illicium v.* decreased the activation of iNOS, which is responsible for inflammatory and atherogenic processes, in a dose-dependent manner in both HASMCs and in aortas of HFD-fed *ApoE*^*−/−*^ mice. Thus, results of the present study suggest that beneficial effects of *Illicium v.* may be mediated through significant attenuation of the iNOS inflammatory response.

We recently reported that major chemical constituent of *Illicium v.* is trans-anethole [1-methoxy-4-(1-propenyl) benzene], which accounts for approximately 85–90 % of the essential oil. Previous reports had indicated that ethanol extracts of *Illicium v.* contain high levels, approximately 2.14 ± 0.01 mg/g, of trans-anethole [28], and the anti-inflammatory and anti-oxidant effects of that compound occur through inhibition of lipid peroxidation [10]. In other studies, trans-anethole was found to decrease production of IL-1β, IL-6, and TARC, to have anti-inflammatory properties in HaCaT cells, and to have anticarcinogenic properties through inhibition of TNF-α-stimulated cellular responses [5, 30]. Hence, trans-anethole may play a substantial role in the action of *Illicium v.* that we have described in our hyperlipidemia model.

We observed iNOS-positive immunostaining and elevated inflammatory cytokine expression, such as IL-1β, Iκκ α/β, and TNF-α, in aortas of HFD-fed *ApoE*^*−/−*^ mice compared to controls, and these effects were practically abolished by *Illicium v.* treatment. Moreover, *Illicium v.* reduced expression of adhesion molecules. It has been reported that atherosclerotic lesion development is accompanied by upregulation of adhesion molecule levels. In hyperlipidemic aortas, inflammation is associated with atherosclerotic plaques in general [19, 21]. Therefore, *Illicium v.* may be a potentially beneficial therapeutic drug that targets the inflammatory cascade critical for pathogenesis of hyperlipidemia-atherosclerosis.

In summary, we have demonstrated in HFD-fed *ApoE*^*−/−*^ mice that *Illicium v.* suppresses aortic atherosclerotic lesion formation by antagonizing changes in body weight, lipid levels, levels of blood pressure, inflammatory cytokines, and adhesion molecules that are mediated through iNOS activation. Beneficial effects of *Illicium v.* may be explained by a significant reduction in the inflammatory response. Therefore, *Illicium v.* is a promising novel therapeutic drug for hyperlipidemia-atherosclerosis treatment.

## Conclusions

We have described an *Illicium v.-*mediated attenuation of atherosclerotic lesion area (size of plaques) and iNOS activation. In addition, we found an inhibition of inflammatory cytokine and adhesion molecule expression, and subsequent blockade of the inflammatory response by the plant extract. These results indicate that *Illicium v.* may be a useful tool for developing new therapeutic opportunities for treatment of hyperlipidemia-atherosclerosis.

## Abbreviations

HASMC: Human aortic smooth muscle cell
TNF-α: Tumor necrosis factor-α
ApoE: Apolipoprotein E
VCAM: Vascular cell adhesion molecule
ICAM: Intercellular adhesion molecule
NF-κB: Nuclear factor kappa B
KIOM: Korea Institute of Oriental Medicine
